# Multiplexed assays of variant effects contribute to a growing genotype–phenotype atlas

**DOI:** 10.1007/s00439-018-1916-x

**Published:** 2018-08-02

**Authors:** Jochen Weile, Frederick P. Roth

**Affiliations:** 1 The Donnelly Centre, University of Toronto, Toronto, ON Canada; 2 Lunenfeld-Tanenbaum Research Institute, Mount Sinai Hospital, Toronto, ON Canada; 3 Department of Molecular Genetics, University of Toronto, Toronto, ON Canada; 4 Department of Computer Science, University of Toronto, Toronto, ON Canada

**Keywords:** Deep mutational scanning, MAVE, Variant effect, VUS, Variants of uncertain significance

## Abstract

Given the constantly improving cost and speed of genome sequencing, it is reasonable to expect that personal genomes will soon be known for many millions of humans. This stands in stark contrast with our limited ability to interpret the sequence variants which we find. Although it is, perhaps, easiest to interpret variants in coding regions, knowledge of functional impact is unknown for the vast majority of missense variants. While many computational approaches can predict the impact of coding variants, they are given a little weight in the current guidelines for interpreting clinical variants. Laboratory assays produce comparatively more trustworthy results, but until recently did not scale to the space of all possible mutations. The development of deep mutational scanning and other multiplexed assays of variant effect has now brought feasibility of this endeavour within view. Here, we review progress in this field over the last decade, break down the different approaches into their components, and compare methodological differences.

## Introduction

Linking genotype to phenotype is a very difficult problem. The parts of the human genome which we understand best are protein-coding genes, yet they only constitute a small fraction of the whole. Impacts of mutations in other functional elements such as splice sites, promoters, or regulatory sequences are more difficult to assay, not to mention the vast stretches of intergenic space. While one might expect a priori that any given intergenic variant is unlikely to bear functional significance, a large number of loci identified as correlated with diseases in genome-wide association studies (GWAS) are found within these regions (Maurano et al. 2012; Edwards et al. 2013). While many of these cases may stem from linkage disequilibrium to coding or splice-altering variants (Taşan et al. 2015), more functions yet unknown may lie hidden within this vast space. Even for protein-coding sequences, the problem is far from simple. Alleles with simple Mendelian behaviour are the exception rather than the rule. Most phenotypes are complex, i.e., they emerge through the interplay of many different genetic or environmental factors. Conversely, many genes are also pleiotropic, i.e., they are involved in more than one mechanism (Chesmore et al. 2018). As a result of this complexity, mutations found in different people may have different quantitative or qualitative effects—phenomena that are correspondingly termed variable expressivity and incomplete penetrance. Similarly, two different mutations within the same coding sequence will often differ by effect. Depending on how the translated protein is affected (e.g., catastrophic folding failure, alteration of a molecular interaction interface or active site, or a subtle change on an unused surface), the effects may differ in severity or in rare cases may even result in the emergence of qualitatively different behaviours.

**Fig. 1:**
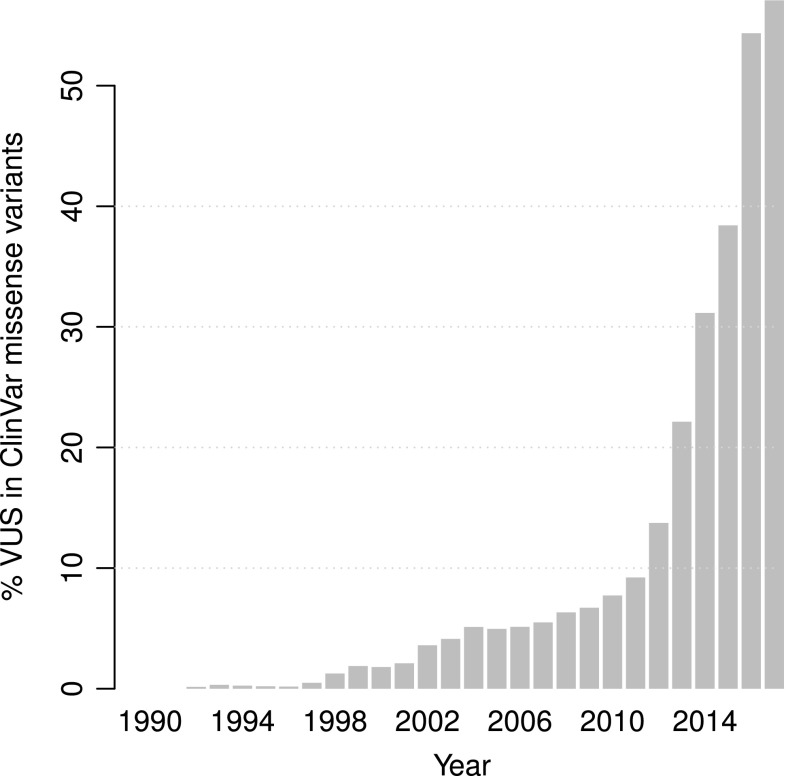
Percentage of variants of uncertain significance (VUS) among missense allele Clinvar records over time from 1990 until 2017

Given the much greater difficulty of interpreting non-coding regions, clinical applications have so far largely concentrated on protein-coding genes. Sequencing panels for known disease-associated genes and even whole-exome sequencing (WES) are widely commercially available. A number of different standards for classifying mutations with respect to their potential health impacts have been proposed; most prominently, the American College of Medical Genetics and Genomics (ACMG) standard (Richards et al. 2015). It defines categories stretching from “pathogenic” to “benign”, including the ‘gray zone’ category of “variant of uncertain significance” (VUS). Even though the mutational landscape for a handful of genes, such as *BRCA1* are explored better than others due to their established relevance and potential for taking clinical action (Cheon et al. 2014), the majority of clinical variants across all genes are currently classified as VUS. In ClinVar alone, VUS make up over 50% of entries for missense variants (Fig. 1), despite ClinVar guidelines that actively discourage submission of unclassified variants. In a recent study using gene panels assessing germline cancer risk loci (Maxwell et al. 2016), over 98% of missense variants were classified as VUS. Not only can these uncertainties burden patients with unnecessary anxiety (Cheon et al. 2014), they also call into question the value of sequencing in the clinic if the majority of findings are not actionable. With increasing use of WES and WGS as opposed to targeted gene panels, this problem is only going to get worse. According to the 1000 Genomes Project data, every person carries 100–400 missense variants that are so rare that they have likely never been seen before in the clinic (The 1000 Genomes Project Consortium 2015). In the absence of the previous observations, they would automatically be added to the long list of VUSs.

Like its sister standards, the ACMG guidelines also recognize different methods of gathering evidence towards a variant’s classification. These can be broadly summarized as (1) frequency of observation in affected or unaffected individuals; (2) laboratory assays; and (3) in silico prediction. Out of these three categories, in silico prediction used to be the only option that easily scaled to cover all possible variants and could be applied proactively. However, it is also considered one of the weakest forms of evidence. Over the last decade, however, a new type of high-throughput laboratory assay has emerged: Multiplexed Assays of Variant Effect (MAVEs) (Starita et al. 2017), which promise to massively increase the scalability of those methods that the ACMG considers in the highest tiers of evidence. In the following, we will first recapitulate some of the more popular in silico approaches and then discuss MAVEs, breaking down the methodological variety in the existing studies, describing some of the newest developments and their implications for the future.

## In silico approaches to variant function assessment

A number of algorithms exist that offer predictions as to the deleteriousness of mutations, with prominent examples including PolyPhen-2 (Adzhubei et al. 2013), SIFT (Ng and Henikoff 2001), and PROVEAN (Choi et al. 2012). PolyPhen-2 employs a simple (naive Bayes) machine learning method based on evolutionary conservation and protein structural features. It uses a set of previously reported pathogenic alleles as a positive training set and differences between human genes and their mammalian homologues as a negative training set. By contrast, SIFT (Sorting Intolerant From Tolerant) only uses evolutionary conservation. The tool uses multiple sequence alignments to calculate position-specific score matrices for each gene which are then normalized and transformed into probability values. PROVEAN (PROtein Variation Effect ANalyzer) similarly only takes into account sequence alignments. However, rather than just computing a position-specific score, PROVEAN calculates the difference in alignment quality between using the wild-type or variant sequence against clusters of homologous sequences. The average distance is then interpreted as indicative of the deleteriousness of the variant.

While the three tools succeed in making good predictions, their reliability is unfortunately still not high enough to serve as a basis of clinical decision making. We and others recently performed an independent comparison of these tools on a set of well-established disease-causing variants as well as rare polymorphisms with no known disease association (Sun et al. 2016). The study examined the trade-off between precision (the fraction of pathogenic variant predictions that were correct) and sensitivity (the fraction of pathogenic variants that were predicted to be pathogenic). A high precision can be considered especially important when considering taking clinical action based on a prediction. When compared at a minimum precision level of 90%, PolyPhen-2 and PROVEAN only reach a sensitivity of 19 and 21%, respectively, while SIFT did not achieve 90% precision at any score threshold. Consistent with these limitations, the ACMG currently considers only cases in which multiple methodologically orthogonal prediction algorithms agree as weak evidence in a supporting role for VUS re-classification (Richards et al. 2015).

## Multiplex assays of variant effect (MAVE)

An alternative to the in silico methods above are functional assays in the laboratory. Such assays are, indeed, useful tools for the classification of variants of uncertain significance. Assessment of the effects of variants observed in the clinic has led to many high-impact discoveries such as drug resistance variants in cancer genes (Solit et al. 2006; Azam et al. 2003; Shah et al. 2002; Kohsaka et al. 2017). However, experimental assays of variant function have generally been ’reactive’, in the sense that measurements are carried out only after (often long after) the first clinical presentation of a variant, owing to the resource- and time-intensive nature of this testing. However, as more variants are discovered, it may be more useful to take a proactive experimental approach: Building an atlas of the functional effects of all possible variants, including those that have never before been observed in a patient. One may object that it would not be economical to screen variants that may never actually be observed in a patient. However, a simple back-of-envelope calculation given the size of the human population and the frequency of de novo mutation (Acuna-Hidalgo et al. 2016) shows that every missense variant that can possibly exist (i.e., it is not fundamentally incompatible with life) can be expected to occur on average in

$$\begin{aligned} \frac{7.6 \times 10^9 \text {humans} \times 0.6 \text { de novo exome SNVs}}{30\text {Mb exome} \times 3 \text { possible SNVs per bp}} \approx 51\, \text {humans}. \end{aligned}$$

Yet, assaying all possible variants in known disease genes would require massive parallelization. Such efforts have recently gained much traction, having their foundations laid in the winter of 2010/11 with three papers by Fowler et al. (2010), Ernst et al. (2010), and Hietpas et al. (2011) that collectively pioneered a technology initially termed Deep Mutational Scanning (DMS). These seminal papers have since inspired a growing number of similar efforts by other groups. While the earliest studies of this kind focused on coding regions, multiple groups have since begun interrogating the effects of non-coding variants, e.g., on promoter activity (Kwasnieski et al. 2012; Maricque et al. 2017), autonomously replicating sequences (Liachko et al. 2013; Hoggard et al. 2016), splicing (Julien et al. 2016; Ke et al. 2018), or the behaviour of RNAs (Li et al. 2016; Puchta et al. 2016). The term “Multiplex Assays of Variant Effect” (MAVE) was coined by Starita et al. (2017) to encompass these high-throughput functional assays for a wider range of variant types.

**Fig. 2:**
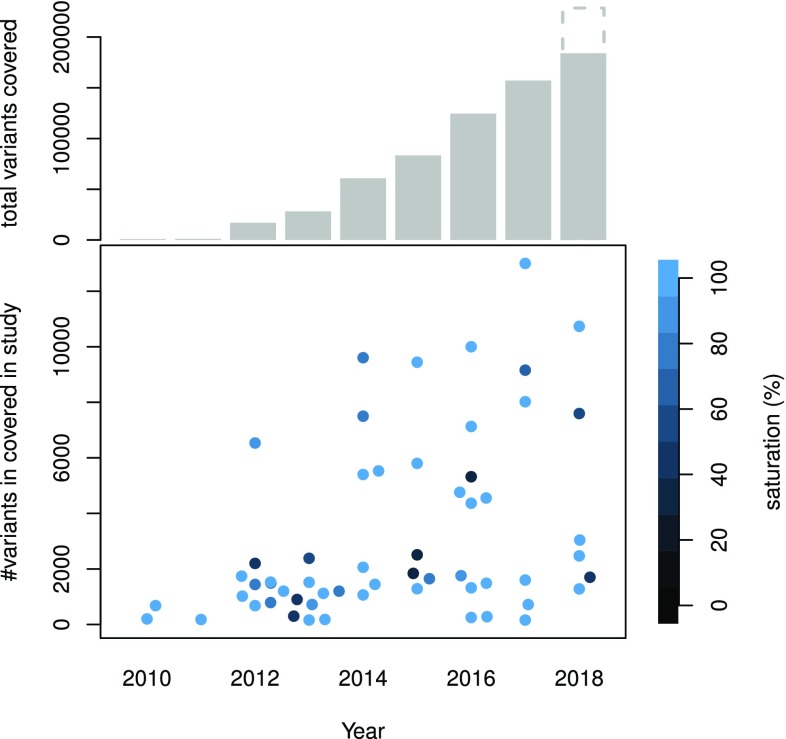
Variant effects covered in MAVE studies. Top: the total number of variant effects covered in MAVE studies up to a given year. For 2018, the solid bar indicates the current state, while the dashed outline represents an extrapolation for the rest of the year. Bottom: the number of variant effects reported in individual studies, where colour indicates the study’s saturation of its respective target space

**Fig. 3:**
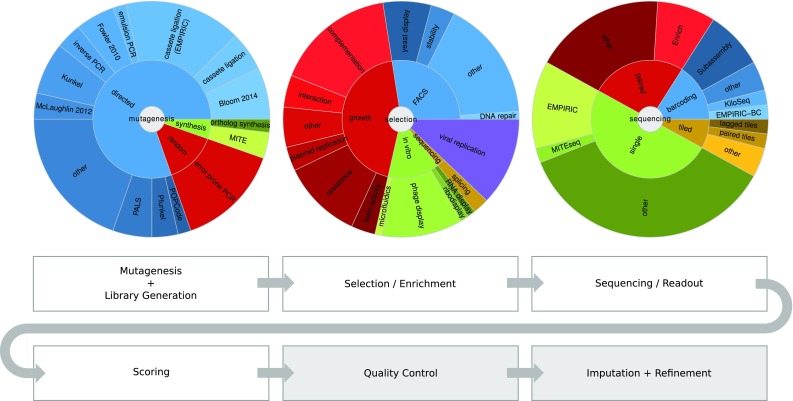
Generalized workflow of a typical MAVE experiment. Steps marked in gray are downstream computational procedures not found in every study, but contribute to the quality of the data. The proportions of studies using different mutagenesis, selection, and sequencing methods are broken down in pie charts. Colors serve to visually differentiate different categories but do not bear meaning

Table 1 lists a selection of MAVE studies and their respective scales, the growth of which is shown in Fig. 2. MAVE screens can be broken down into a number of experimental and computational components: (1) mutagenesis and library creation; (2) selection of functional variants; (3) sequencing of the selected and control populations; (4) scoring and computational analysis (see Fig. 3). In the following sections, we will review the different previous implementations of these components in detail.

**Table 1:** List of MAVE studies, their respective target spaces, and achieved levels of coverage

Reference	Target	Search space	Coverage (%)
Fowler et al. (2010)	YAP65	WW domain	$\sim 100$
Ernst et al. (2010)	Synthetic PDZ domain	10 AAs	$\sim 100$
Hietpas et al. (2011)	Hsp90	9 AAs	$\sim 100$
Fujino et al. (2012)	Fab antibody fragment	Fragment	79
Adkar et al. (2012)	Ccdb	whole protein	$< 74$
McLaughlin et al. (2012)	PSD95	PDZ domain	$\sim 100$
Schlinkmann et al. (2012)	GPCR	Whole protein	$\sim 90$
Whitehead et al. (2012)	Synthetic protein	51AA (whole protein)	99
Traxlmayr et al. (2012)	IgG1	CH2/CH3 domains	$< 50$
Araya et al. (2012)	YAP65	WW domain	$\sim 100$
Deng et al. (2012)	TEM1	Whole protein	$\sim 80$
Kwasnieski et al. (2012)	Rhodopsin promoter	52 bp cis-regulatory element	$\sim 100$
Wu et al. (2013)	Neuraminidase	SNP accessible	$<50$
Roscoe et al. (2013)	Ubiquitin	Whole protein	$\sim 95$
Starita et al. (2013)	Ub.E3 E4B	Whole protein	$\sim 50$
Procko et al. (2013)	Synthetic protein	60 AA	$\sim 100$
Tinberg et al. (2013)	Synthetic protein	40AA	90
Jiang et al. (2013)	Hsp90	Substrate binding loop	$\sim 100$
Kim et al. (2013)	Mat alpha	Degron region	$< 50$
Melamed et al. (2013)	Pab1	RRM domain	$\sim 90$
Forsyth et al. (2013)	Antibody for EGFR	Whole protein	$\sim 99$
Jacquier et al. (2013)	TEM1	whole protein	64
Hietpas et al. (2013)	Hsp90	pos. 528–590	$\sim 100$
Liachko et al. (2013)	ARS1	100 bp	$\sim 100$
Wagenaar et al. (2014)	BRAF	77 AAs	99.65
Firnberg et al. (2014)	TEM1 $\beta$-lactamase	Whole protein	$\sim 95$
Olson et al. (2014)	G-protein (GB1)	IgG-binding domain	$\sim 95$
Melnikov et al. (2014)	APH(3’)II (kinase)	Whole protein	$\sim 100$
Bloom (2014)	Influenza nucleoprotein	Whole protein	$> 75$
Thyagarajan and Bloom (2014)	Influenza hemagglutinin	Whole protein	$\sim 85$
Qi et al. (2014)	NS5A	IA domain	$\sim 100$
Roscoe and Bolon (2014)	Ubiquitin	Whole protein	$\sim 95$
Reich et al. (2015)	Bcl-$x_L$ ligands	Peptide library	N/A
Stiffler et al. (2015)	TEM1 $\beta$-Lactamase	Whole protein	$\sim 100$
Doud et al. (2015)	Influenza nucleoprotein	Whole protein	$\sim 100$
Kitzman et al. (2015)	Gal4	DB domain	$\sim 99$
Starita et al. (2015)	BRCA1	RING domain	$\sim 80$
Rockah-Shmuel et al. (2015)	M.HaeIII	Whole protein	38
Wu et al. (2015)	PA	Whole protein	94
Mishra et al. (2016)	Hsp90	ATPase domain	$\sim 99$
Doud and Bloom (2016)	Hemagglutinin	Whole protein	$< 97$
Mavor et al. (2016)	Ubiquitin	Whole protein	$\sim 99$
Majithia et al. (2016)	PPAR$\gamma$	Whole protein	$\sim 99$
Julien et al. (2016)	FAS/CD95	Exon 6	95
Li et al. (2016)	tRNA Arg-CCU	whole gene	$\sim 100$
Sarkisyan et al. (2016)	GFP	whole protein	$\sim 100$
Tripathi et al. (2016)	CcdB	whole protein	87
Puchta et al. (2016)	snoRNA U3	pos. 7-333	$\sim 100$
Brenan et al. (2016)	Mapk1/Erk2	Whole protein	99
Steinberg and Ostermeier (2016)	TEM1	Whole protein	39–50
Hoggard et al. (2016)	miniARS317/301	$153+135$ bp	$\sim 100$
Ma et al. (2017)	BCR-ABL	8AAs	$\sim 100$
Matreyek et al. (2017)	GFP	whole protein	$\sim 60$
Klesmith et al. (2017)	TEM1,LGK	whole proteins	$\sim 70$
Chan et al. (2017)	IGPS	8 $\beta$-strands	$\sim 95$
Bandaru et al. (2017)	H-Ras	pos. 2–166	$\sim 100$
Weile et al. (2017)	UBE2I,SUMO1,TPK1,CALM1/2/3	whole proteins	100
Mighell et al. (2018)	PTEN	Whole protein	$\sim 95$
Plesa et al. (2018)	PPAT	Whole protein	$\sim 95$
Matreyek et al. (2018)	PTEN,TPMT	Whole protein	$\sim 60$
Ke et al. (2018)	DHFR	exon 5	$\sim 100$
Starita et al. (2018)	BRCA1	302 AAs	$< 50$
Kotler et al. (2018)	TP53	DNA-binding domain	$\sim 85$

### Mutagenesis approaches

A variety of saturation mutagenesis methods have previously been applied in MAVE studies; some more technically challenging than others. The simplest method is error-prone PCR amplification (Cadwell and Joyce 1994; Mohan et al. 2011). While this has the advantage of being an inexpensive and facile procedure, it will almost exclusively result in the generation of point mutations and as such will not generate all possible amino acid replacements. One may argue that the evaluation of VUS does not require insight into amino acid substitutions that cannot be achieved by a single-nucleotide change, as they are unlikely to occur in the clinic. However, the preference for transitions over transversions in many error-prone PCR protocols can lead to uneven representations of variants, so that codon-level mutagenesis can lead to more even representation amongst those missense variants that are achievable by single nucleotide change. In addition, multiple nucleotide changes do occur within single codons, a non-negligible 2% of the time (Kaplanis et al. 2018). Moreover, exploring all possible amino acid changes offers the potential for valuable insights into what biochemical properties may be most important for each amino acid at each position.

Another set of methods often employed are scaled-up versions of site-directed mutagenesis approaches (Hutchison et al. 1978; Seyfang and Jin 2004; Firnberg and Ostermeier 2012), with one popular example being Kunkel mutagenesis (Kunkel 1985). The Kunkel approach uses a strain of *E. coli* that has been modified to produce high levels of uridine and lacks the ability to excise these bases from DNA. A phage vector carrying the desired template sequence is transfected into the cells resulting in its replication with a high uracil incorporation rate. The thus-uracilated plasmid can then be used as a template for primer extension, with primers containing the mutations of interest, and subsequently introduced into wild-type *E. coli* which will degrade the uracilated template, thus enriching the mutant copies. A number of derivatives of Kunkel mutagenesis have since been developed to bring its output to scale supporting saturated libraries, most notably Pfunkel (Firnberg and Ostermeier 2012). To address the full spectrum of amino acids at a given position, oligonucleotides carrying degeneracy codons (Pal and Fellouse 2005) are often used. Particularly popular is the use of NNK and NNS degeneracies, which have long been used in biochemistry (Scott and Smith 1990; Barbas et al. 1992). Here, S denotes either Guanine or Cytosine, and K denotes either Guanine or Thymine in the third position of the degenerate codon. Either of these options only enables 32 out of all 64 possible codons, covering all 20 possible amino acids while avoiding two of the three possible stop codons (TGA and TAA). An alternative to degeneracy codes is the use of custom oligonucleotide arrays covering all possible (or desired) options of codon changes explicitly (Kitzman et al. 2015). While this option allows for the precise control of desired mutations, it is currently too expensive to be applicable for more than a handful of genes at a time.

Another saturation mutagenesis method often applied in Deep Mutational Scanning is EMPIRIC (“Extremely Methodical and Parallel Investigation of Randomized Individual Codons”) (Hietpas et al. 2011). In this method, rather than using PCR amplification, oligonucleotide cassettes carrying the variants of interest are directly ligated at the appropriate positions. This is achieved by designing the underlying vector, such that it omits the cassette sequence. Instead, it carries a restriction site at the equivalent position, which can be cut to create sticky ends. Pairs of oligos carrying the variants of interest can be synthesized, such that they can assemble into a fitting cassette that integrates with the vector. EMPIRIC is one example of a mutagenesis method that was explicitly developed to be used in Deep Mutational Scanning. Another example is PALS (“Programmed ALlelic Series”) (Kitzman et al. 2015), which aims to limit the number of amino acid changes per library clone to only one. Oligos carrying the variants of interest are annealed to uracilated templates and linearly amplified with strand-displacing polymerase. In a second step, the template is degraded using uracil-DNA glycosylase and an antisense strand is generated in a second linear amplification step. The product is denatured and yet again hybridized with uracilated template allowing it to be extended towards the other end of the template. Finally, the template is degraded again and the now full-length mutagenized strands are amplified.

Yet another approach, which recently gained popularity, is dubbed “inverse PCR” (Jain and Varadarajan 2014). This method uses circular templates and pairs of oligos, one of which carries the mutagenic degenerate sequence, while the other points directly away from it. This primer setup appears like a directional inversion of that used in a regular PCR, thus lending the method its name. A first amplification step produces a set of linear products which serve as templates for the second, exponential amplification step, after which the final product is circularized. The authors compared the method to similar approaches using overlapping primers and found the inverse PCR method to display superior efficiency. This approach has become popular recently, and multiple MAVE maps have relied on it (Puchta et al. 2016; Matreyek et al. 2018).

A more recent development is “POPCode” (Weile et al. 2017), which expands upon the site-directed approach described by Seyfang and Jin (2004). Here, a set of oligos carrying all possible codon replacements are designed, such that their melting temperatures are uniform. They are hybridized to a uracilated template, in similar fashion to the PALS approach; however, they are allowed to directly compete with each other, enabling either single or multiple variants per molecule, depending on oligonucleotide concentrations. Non-strand-displacing polymerase is used to fill the gaps and seal the remaining nicks, followed by the degradation of the template using uracil-DNA glycosylase. A useful feature of this approach is the availability of a webtool that automates POPCode oligo design, such that each oligo arm surrounding the degeneracy has a similar melting temperature (Weile et al. 2017).

In addition to the various mutagenesis methods discussed here, it may be noted that complete variant libraries are also recently becoming commercially available via gene synthesis (Kosuri et al. 2010). A current limitation is the rate of point mutations and indels (Plesa et al. 2018), which makes it inappropriate for achieving frameshift-free coding regions with  1 aa change per clone for longer proteins. However, it is possible that, with increased interest in gene synthesis applications, these options may become more accurate and economical in the future.

Finally, with the rise of CRISPR/Cas9 and other gene editing tools, new methods are emerging that are able to introduce variants directly into endogenous gene loci, at efficiencies which are beginning to allow saturation mutagenesis. Findlay et al. (2014) first demonstrated this idea on a small scale, mutagenizing a small range of codons in BRCA1. A large-scale implementation of this idea is soon to be published (Findlay et al. 2018). Although this is currently more resource-intensive than introduction of an mutagenized library generated ex vivo, the advantages of studying variation in the context of the native gene locus are potentially great. For example, the function of variants that depend on the action of distal enhancers would be missed using mutagenized constructs introduced at a safe harbor site that is far from the endogenous locus.

### Selection approaches

The most central component of a MAVE study is the selection process. The selection schemes used in previous studies can be sorted into four broad categories: (1) in vitro display methods (such as phage display or ribodisplay); (2) competition-based methods that couple a protein property under investigation (such as molecular interactions, toxicity, or overall functionality) to host cell fitness; (3) cell sorting based on fluorescence-labeled reporters; (4) transcript-abundance-based methods.

Phage display (Smith 1985) and ribodisplay (Mattheakis et al. 1994) couple the genetic information of each given variant to the physical protein itself and select according to the protein’s ability to bind to a fixed interactor. In phage display, this is achieved by the protein being displayed on the surface of a phage that contains the corresponding gene, while ribodisplay stalls a cluster of ribosomes on the variant mRNA with the corresponding protein still attached. Variants that are unable to bind to the interactor-coated surface are washed away and thus depleted. This can be done in multiple rounds, as the associated genetic information can be replicated again after selection (via viral propagation in bacteria for phage display or via PCR in ribodisplay). Fowler et al. (2010) employed phage display in their seminal Deep Mutational Scanning study of the binding of the YAP65-WW domain to its cognate peptide target. However, since display methods are only feasible for small proteins or fragments thereof, more recent studies have instead employed more scalable methods.

The most frequently applied selection mechanisms are fitness-based. In these cases, a particular property of the variant protein is coupled to its host cell’s ability to thrive in competitive growth. Of these methods, functional complementation (Lee and Nurse 1987; Osborn and Miller 2007) and Yeast-2-Hybrid (Y2H) (Fields and Song 1989) are among the most frequently applied. While complementation couples fitness to a protein’s overall ability to perform its biological role in a model organism, Y2H couples fitness to the ability of the protein to maintain a specific protein–protein interaction.

The largest share of growth-based selection methods in MAVE studies employs functional complementation, and most use the yeast *Saccharomyces cerevisiae* as their model system (see Fig. 3). The assay is based on the premise that some human genes can be used to rescue the deletion of their orthologues in yeast. That is, a fitness defect resulting from the inactivation of the yeast gene is alleviated by the artificial expression of the human gene. Therefore, any relative changes in fitness upon expressing a variant of the human gene can be interpreted as the variant’s effect on the protein’s overall ability to function. We and others recently examined the applicability of functional complementation in yeast to the assessment of disease variants (Sun et al. 2016). This study found that functional complementation assays in yeast offered sensitive and accurate predictions despite yeast and humans being diverged by $\sim$ 1 billion years. Indeed, yeast complementation outperformed in silico methods like PolyPhen-2 and PROVEAN in terms of disease variant prediction by a wide margin. At a threshold of 90% precision (as discussed in “In silico approaches to variant function assessment”), the complementation assay achieved a sensitivity of over 60%, as compared to 19 and 21% for the two in silico methods, respectively. It is consistent with these findings that the ACMG considers functional assays among the strongest sources of evidence for variant classification (Richards et al. 2015).

A limitation of functional complementation in yeast is that currently only $\sim 200$ human disease-implicated genes have been found to be amenable to the assay (Sun et al. 2016). However, the existence of synthetic lethal genetic interactions for many yeast genes may allow for the design of strains with sensitized backgrounds providing new complementation assays. In addition, CRISPR screens have in recent years revealed many genes for which growth phenotypes exist directly in human cell lines (Hart et al. 2015; Blomen et al. 2015; Wang et al. 2014); opening the possibility of performing functional complementation directly in these cell lines. A number of studies have since exploited the possibility of complemention assays directly in human cells (e.g., Wagenaar et al. 2014; Qi et al. 2014; Brenan et al. 2016). However, while future variant analysis is likely to trend towards complementation in mammalian cell models as opposed to yeast, the latter assays are not obsolete. Sun et al. (2016) found that a selectable phenotype that is potentially compatible with MAVE using human cell-based complementation has been identified for less than half of all human disease genes. Thus, for many disease genes, yeast-based complementation or Y2H may be the only viable option for a MAVE.

A popular condition-dependent extension to complementation is selection for drug resistance (Wu et al. 2013; Wagenaar et al. 2014), but other fitness-based selection methods have been used in MAVEs as well. For example, Adkar et al. (2012) used the toxicity of CCDB in *E. coli*, while Kim et al. (2013) select according to degron activity by fusing the degron to an auxotrophic marker. Finally, a number of MAVE studies have been performed on viral genes, by selecting for virus propagation efficiency (Bloom 2014; Thyagarajan and Bloom 2014).

Another popular growth-based assay is Yeast-2-Hybrid (Y2H). It is a binary protein interaction assay also performed within the yeast *S. cerevisiae*. Y2H is based on the reconstitution of two fragments of the transcription factor Gal4 fused to two proteins of interest. A successful interaction of the two proteins allows Gal4 to induce the expression of a reporter; usually, an auxotrophy marker. When comparing different variants of the same protein interacting with the same partner, reporter expression has even been shown to be proportional to binding affinity (Yang et al. 1995). This proportional relationship allows for quantitative interpretation of Y2H results under these specific circumstances.

One objection to the use of Y2H as an assay for variant function assessment is that it does not measure all aspects of a protein’s functionality, but rather only its ability to physically associate with a given interaction partner. However, in addition to detecting variants that specifically affect binding, e.g., via changes to the binding interface, this approach should also detect many other variants that broadly impact protein function, e.g., those that cause protein mis-folding or instability. Indeed, in a recent examination of the Y2H performance of common disease-associated variants, we found that approximately two out of three disease variants in proteins with multiple interaction partners lose some or all of their protein interactions (Sahni et al. 2015).

Another selection mechanism is the use of fluorescence-activated cell sorting (FACS) (Julius et al. 1972). Here, surface markers for which abundance is proportional to the activity of the studied protein are targeted with fluorescently labeled antibodies, such that cells can be sorted accordingly, as has been performed by Schlinkmann et al. (2012) and Majithia et al. (2016). FACS-based selection has also been used to gain read-outs of protein stability and abundance (Matreyek et al. 2018).

In addition to assays that measure general properties of proteins, more specialized methods also exist, which assess specific molecular functions. For example, Starita et al. (2015) developed an assay that quantifies the ubiquitination activity of BRCA1’s RING domain. Most recently, they also developed an assay to test the same protein’s DNA-repair activity (Starita et al. 2018).

In terms of selection for the properties of non-coding regions, a number of technologies have been developed. Massively Parallel Reporter Assays (MPRAs) (Kwasnieski et al. 2012; Maricque et al. 2017) for example place libraries of mutagenized promoter sequences upstream of barcoded regions, the expression of which is measured using RNA-Seq, while the initial abundance of corresponding cells is measured by DNA sequencing of the same loci. The ratio of RNA-Seq to DNA-Seq reads can then be used to calculate the effect of promoter variant on expression. Similarly, splicing assays such as employed by Julien et al. (2016) or Ke et al. (2018) also use RNA-Seq to measure the fraction of transcripts in which the exon of interest is spliced in.

### Sequencing strategies

The experimental step immediately following selection in a MAVE experiment is sequencing. Next-generation sequencing technology can be considered the key technological advance that made Deep Mutational Scanning possible. Many studies use a fairly simple approach by performing deep shotgun sequencing of the library (Ernst et al. 2010; Hietpas et al. 2011; Fujino et al. 2012). However, a major problem with this approach is that, without knowing which reads originate from which DNA molecule, each read can only be considered by itself, making it difficult to distinguish real mutations from sequencing error. To address this problem, different solutions have emerged. In cases where the amplicon is short enough, paired-end ‘duplex’ sequencing can be exploited to use information from both strands for variant calling. In the simplest case, this is achieved by requiring both reads to agree on the base call in question, as in the case of Whitehead et al. (2012) and Weile et al. (2017). A less stringent, but potentially more sensitive alternative as used by Fowler et al. (2010) is to perform Bayesian inference on the quality scores associated with the base calls in each read pair. This way, a variant may still be identified if one of the two reads reported a wild-type base call with low confidence.

Where the length of the nucleotide sequence in question exceeds the read-length capabilities of short-read sequencing technologies, other strategies are required. A notable borderline case can be found in Olson et al. (2014) where only a partial overlap between read pairs was achieved and variant calls outside of the overlap region were of lower quality. Other studies have used more involved approaches. A popular paradigm is the association of molecular barcodes with each clone within the MAVE library. While this simplifies the readout of the experiment (as only the barcodes need to be sequenced and counted), it adds the requirement of identifying which barcode belongs to which genotype. In most cases, this is addressed using “subassembly” (Hiatt et al. 2010), a high-throughput amplicon sequencing approach based on attaching random tags to amplicons. The DNA is then amplified, sheared, and ligated to adapters, so that paired-end sequencing can be used to identify the random tag together with each read. This allows reads to be sorted according to which original tagged molecule they belong, which, in turn, enables separate assemblies for each input molecule to be computed. The resulting high-quality virtual reads are long enough to cover both ORF and barcode locus. Another subassembly approach is “KiloSeq” (Weile et al. 2017) which works using an array-based format where well-specific tags are attached to the amplicons, followed by Tn5 tagmentation and re-amplification of tag-bearing fragments. Another barcode-based method, called EMPIRIC-BC was described by Mavor et al. (2016), where the amplicon in question was short enough not to require subassembly. Here, a long read can cover the entire ORF, while a second, short read can identify the barcode.

An alternative approach to covering longer stretches of DNA is to subdivide them into smaller regions that can be sequenced separately from each other. For example, Doud and Bloom (2016) amplify each region with primers carrying random tags. This way, if multiple reads contain the same tag, they are highly likely to originate from PCR copies of the same original molecule and can be used to make more accurate variant calls. This approach is often dubbed “tag-clustering”. While this approach has the advantage of being less labour-intensive than barcoding each individual clone in the MAVE library, it can only detect variants co-occurring within the same region of the sequence. Thus the library must be designed in such a way that either only a single mutation occurs within each clone or that it is large enough that effects of many background variants are averaged out. This approach can also be used in combination with duplex sequencing, as performed in Weile et al. (2017), where it is called “DMS-TileSeq”.

In a benchmark study, Zhang et al. (2016) evaluated some of these approaches. They found that duplex sequencing decreases the rate of transition and transversion base-calling errors tenfold while decreasing indel errors by 100-fold. By contrast, tag clustering lowered transition and transversion errors 20 fold, but had a little impact on indel errors. A combination of both approaches in which tagged reads are first compared against their paired partners and then clustered is found to perform best. The authors also examined the effect of quality score filtering. While this method had moderate impact when applied to raw reads, read pairing and tag clustering benefited little from it.

### Computational analysis

Most MAVE studies use custom scripts to process the sequencing readout and calculate the selection advantage for each variant. Nonetheless, a few published software packages exist. The EMPIRIC mutagenesis and screening method provides its own software package for data processing (Hietpas et al. 2011), though it is not generally applicable to other MAVE methods. The dms_tools package (Bloom 2015) offers the same services, but is tailored more towards methods using regionally focused sequencing. Finally, Enrich (Fowler et al. 2011) offers a generalized solution applicable to most DMS frameworks. A second version that adds a more sophisticated statistical analysis including the assessment of measurement confidence levels  (Rubin et al. 2016).

The DMS-BarSeq and DMS-TileSeq methods used in Weile et al. (2017) also come with publicly available analysis pipelines. Most importantly, they offer imputation of missing values using machine learning. A Random Forest model (Breiman 2001) was created using physicochemical and structural features of the affected amino acids as well as position-specific biases of the existing map, yielding surprisingly accurate predictions that surpassed those of Polyphen-2 (Adzhubei et al. 2013) and PROVEAN (Choi et al. 2012). This most recent innovation has since been adapted for use with different predictive features by Mighell et al. (2018).

Going beyond the scope of predicting the effects of variants omitted within a mapped gene, Gray et al. (2018) applied machine learning to extrapolating maps for new genes. Using a gradient-boost model (Friedman 2001) trained on similar features as the imputation method from Weile et al. (2017), they implemented a new cross-validation scheme that swaps out whole proteins to be more sensitive towards the detection of overfitting. While predictions were generally more reliable than established computational predictors, accuracy was highly variable across proteins, with some performing better than others. This behaviour may be alleviated as more DMS data sets become available for training.

Beyond the potential utility of the variant effect maps in the clinic, they also lend themselves to extract new insights from computational biology. Bloom (2017) recently developed a method to detect signatures of evolutionary selection within these maps far exceeding the sensitivity of comparing orthologous sequences alone. Meanwhile, Wu et al. (2016) developed a method to calculate direct estimates for the folding energy effects of variants by examining their intragenic genetic interactions within variant effect maps.

## Conclusion

Since their inception in 2010, MAVEs have produced a steadily increasing wealth of variant effect maps. Recent years have seen an increasing trend of targeting clinically relevant genes. The utility of these maps towards the eventual goal of clinical variant assessment has been demonstrated in multiple studies (Starita et al. 2015; Majithia et al. 2016; Weile et al. 2017). Since then, an arsenal of different methodologies have been developed to capture a wider spectrum of sequence types and functions. In addition, new computational methods continue to improve the quality and reliability of the data produced.

However, a number of issues are still apparent. Many of the studies listed in Table 1 do not make their data easily available. While some provide full access to final results, some only provide raw data in the NCBI short-read archive (SRA), the majority require interested parties to contact the authors personally. There is a clear need for an open data repository that makes MAVE data available to the public and allows for downstream probabilistic integration and analysis. Similarly, the issue of reagent availability remains as a challenge. In most cases, the saturation mutagenesis libraries generated are not made available via common repositories. Furthermore, due to the large diversity of methodologies employed, libraries cannot generally be expected to be compatible across platforms.

Another complicating factor is the fact that the assays underlying different MAVE studies are quite diverse and measure different aspects of a protein’s behaviour. As a consequence, they cannot be easily compared with each other. In addition, the achieved coverage of possible amino acid changes varies from map to map. Finally, many maps do not control the quality of measurements. Therefore, the confidence levels underlying different parts of these maps are often unknown. While generalized frameworks have been proposed that would increase the potential comparability and interpretability across maps (Rubin et al. 2016; Weile et al. 2017), they are not implemented by most studies. Here, a centralized repository could also be of help, as it could serve as a basis for re-analysis of data with the latest tools.
